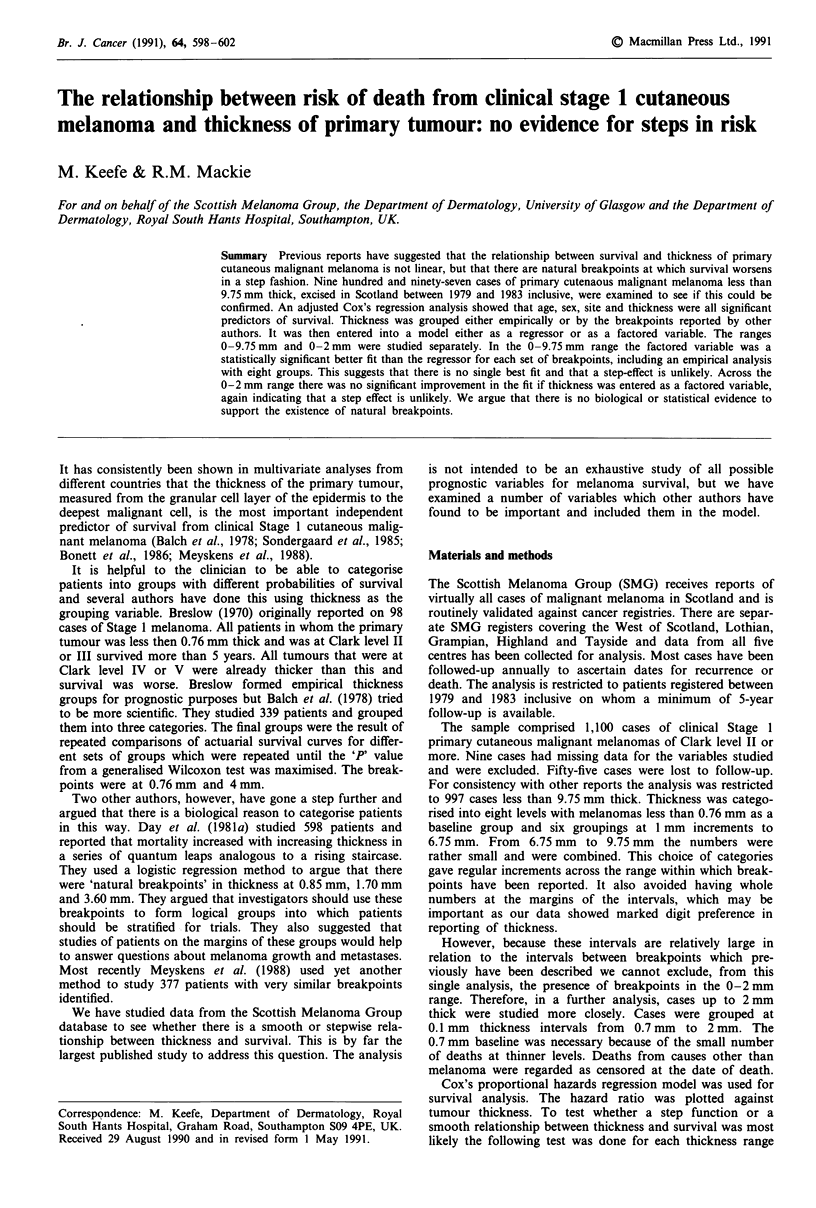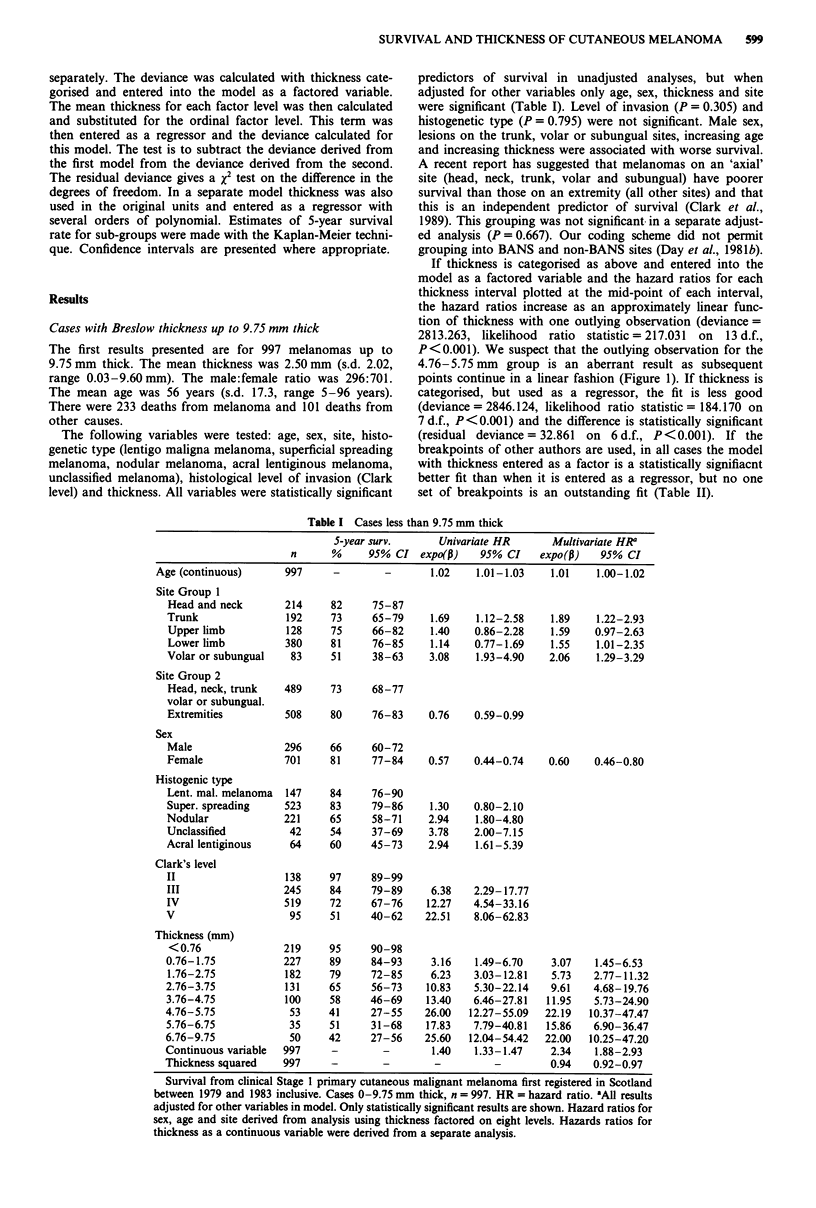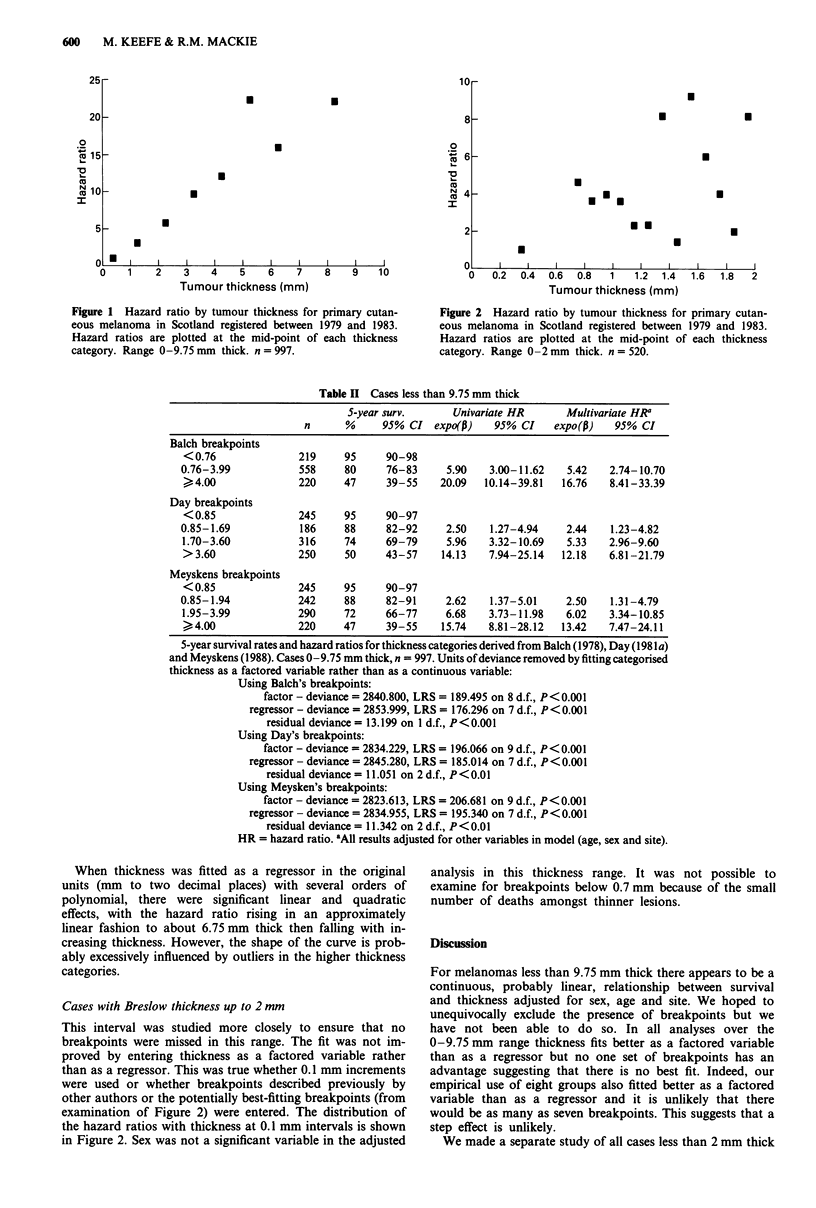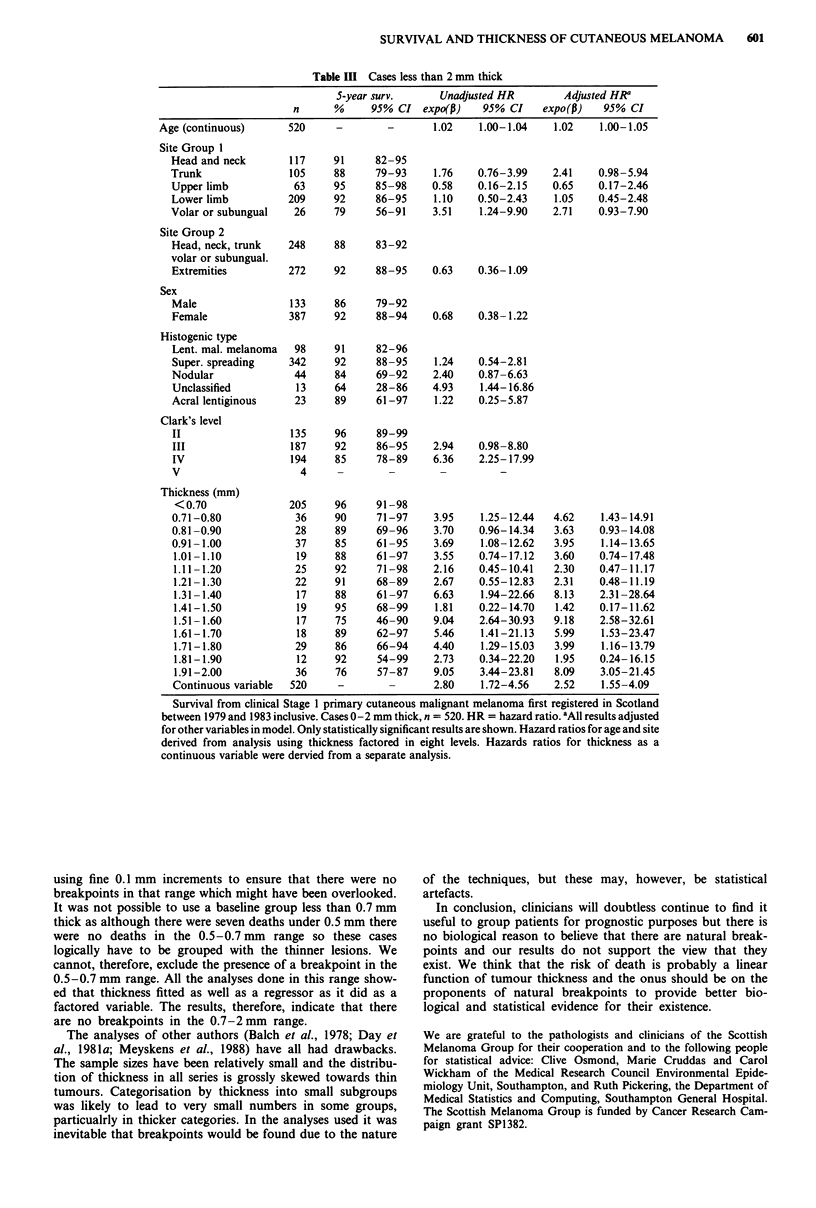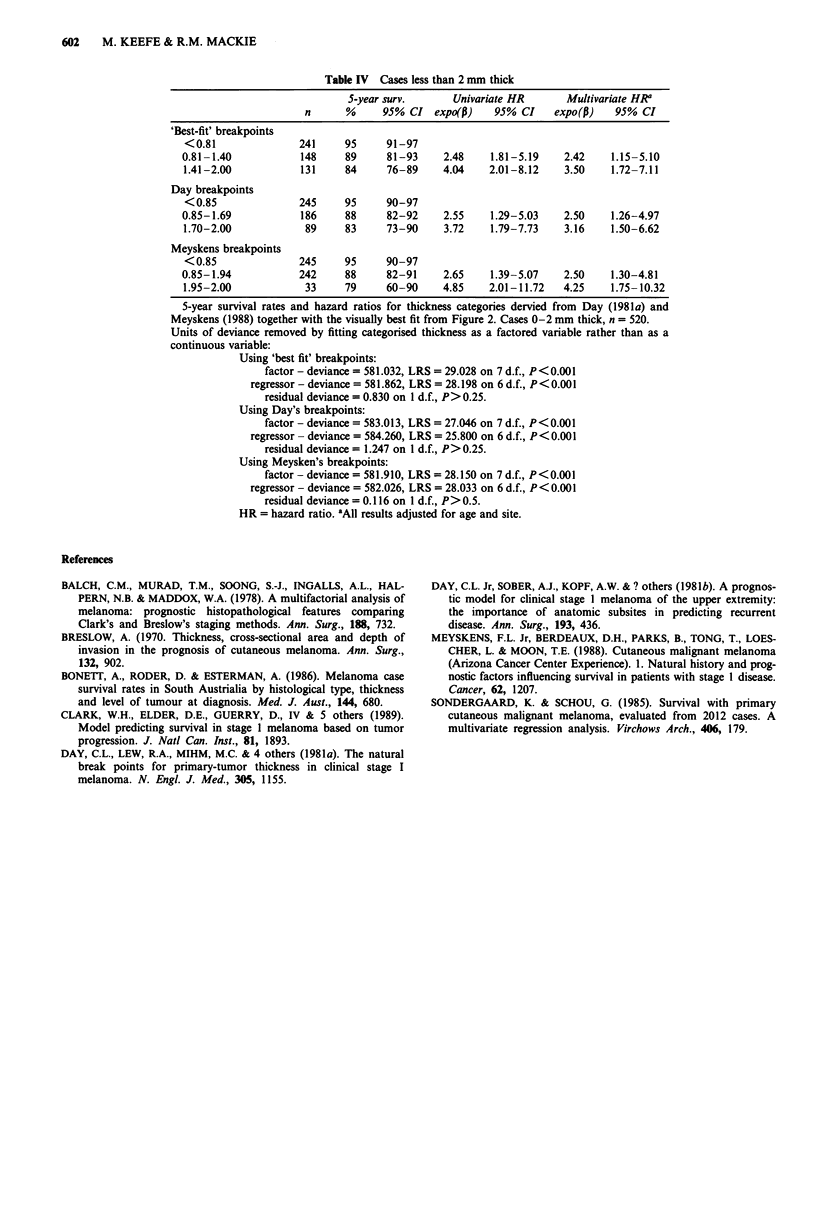# The relationship between risk of death from clinical stage 1 cutaneous melanoma and thickness of primary tumour: no evidence for steps in risk. Scottish Melanoma Group.

**DOI:** 10.1038/bjc.1991.357

**Published:** 1991-09

**Authors:** M. Keefe, R. M. Mackie

**Affiliations:** Department of Dermatology, University of Glasgow, UK.

## Abstract

Previous reports have suggested that the relationship between survival and thickness of primary cutaneous malignant melanoma is not linear, but that there are natural breakpoints at which survival worsens in a step fashion. Nine hundred and ninety-seven cases of primary cutaneous malignant melanoma less than 9.75 mm thick, excised in Scotland between 1979 and 1983 inclusive, were examined to see if this could be confirmed. An adjusted Cox's regression analysis showed that age, sex, site and thickness were all significant predictors of survival. Thickness was grouped either empirically or by the breakpoints reported by other authors. It was then entered into a model either as a regressor or as a factored variable. The ranges 0-9.75 mm and 0-2 mm were studied separately. In the 0-9.75 mm range the factored variable was a statistically significant better fit than the regressor for each set of breakpoints, including an empirical analysis with eight groups. This suggests that there is no single best fit and that a step-effect is unlikely. Across the 0-2 mm range there was no significant improvement in the fit if thickness was entered as a factored variable, again indicating that a step effect is unlikely. We argue that there is no biological or statistical evidence to support the existence of natural breakpoints.


Br. J. Cancer (1991), 64, 598-602 © Macmillan Press Ltd., 1991

### The relationship between risk of death from clinical stage 1 cutaneous melanoma and thickness of primary tumour: no evidence for steps in risk

M. Keefe & R.M. Mackie

*For and on behalf of the Scottish Melanoma Group, the Department of Dermatology, University of Glasgow and the Department of Dermatology, Royal South Hants Hospital, Southampton, UK.*

**Summary** Previous reports have suggested that the relationship between survival and thickness of primary cutaneous malignant melanoma is not linear, but that there are natural breakpoints at which survival worsens in a step fashion. Nine hundred and ninety-seven cases of primary cutenaous malignant melanoma less than 9.75 mm thick, excised in Scotland between 1979 and 1983 inclusive, were examined to see if this could be confirmed. An adjusted Cox's regression analysis showed that age, sex, site and thickness were all significant predictors of survival. Thickness was grouped either empirically or by the breakpoints reported by other authors. It was then entered into a model either as a regressor or as a factored variable. The ranges 0-9.75 mm and 0-2 mm were studied separately. In the 0-9.75 mm range the factored variable was a statistically significant better fit than the regressor for each set of breakpoints, including an empirical analysis with eight groups. This suggests that there is no single best fit and that a step-effect is unlikely. Across the 0-2 mm range there was no significant improvement in the fit if thickness was entered as a factored variable, again indicating that a step effect is unlikely. We argue that there is no biological or statistical evidence to support the existence of natural breakpoints.

It has consistently been shown in multivariate analyses from different countries that the thickness of the primary tumour, measured from the granular cell layer of the epidermis to the deepest malignant cell, is the most important independent predictor of survival from clinical Stage 1 cutaneous malignant melanoma (Balch *et al.*, 1978; Sondergaard *et al.*, 1985; Bonett *et al.*, 1986; Meyskens *et al.*, 1988).

It is helpful to the clinician to be able to categorise patients into groups with different probabilities of survival and several authors have done this using thickness as the grouping variable. Breslow (1970) originally reported on 98 cases of Stage 1 melanoma. All patients in whom the primary tumour was less then 0.76 mm thick and was at Clark level II or III survived more than 5 years. All tumours that were at Clark level IV or V were already thicker than this and survival was worse. Breslow formed empirical thickness groups for prognostic purposes but Balch *et al.* (1978) tried to be more scientific. They studied 339 patients and grouped them into three categories. The final groups were the result of repeated comparisons of actuarial survival curves for different sets of groups which were repeated until the '*P*' value from a generalised Wilcoxon test was maximised. The breakpoints were at 0.76 mm and 4 mm.

Two other authors, however, have gone a step further and argued that there is a biological reason to categorise patients in this way. Day *et al.* (1981*a*) studied 598 patients and reported that mortality increased with increasing thickness in a series of quantum leaps analogous to a rising staircase. They used a logistic regression method to argue that there were 'natural breakpoints' in thickness at 0.85 mm, 1.70 mm and 3.60 mm. They argued that investigators should use these breakpoints to form logical groups into which patients should be stratified for trials. They also suggested that studies of patients on the margins of these groups would help to answer questions about melanoma growth and metastases. Most recently Meyskens *et al.* (1988) used yet another method to study 377 patients with very similar breakpoints identified.

We have studied data from the Scottish Melanoma Group database to see whether there is a smooth or stepwise relationship between thickness and survival. This is by far the largest published study to address this question. The analysis is not intended to be an exhaustive study of all possible prognostic variables for melanoma survival, but we have examined a number of variables which other authors have found to be important and included them in the model.

Correspondence: M. Keefe, Department of Dermatology, Royal South Hants Hospital, Graham Road, Southampton S09 4PE, UK.
Received 29 August 1990 and in revised form 1 May 1991.

### Materials and methods

The Scottish Melanoma Group (SMG) receives reports of virtually all cases of malignant melanoma in Scotland and is routinely validated against cancer registries. There are separate SMG registers covering the West of Scotland, Lothian, Grampian, Highland and Tayside and data from all five centres has been collected for analysis. Most cases have been followed-up annually to ascertain dates for recurrence or death. The analysis is restricted to patients registered between 1979 and 1983 inclusive on whom a minimum of 5-year follow-up is available.

The sample comprised 1,100 cases of clinical Stage 1 primary cutaneous malignant melanomas of Clark level II or more. Nine cases had missing data for the variables studied and were excluded. Fifty-five cases were lost to follow-up. For consistency with other reports the analysis was restricted to 997 cases less than 9.75 mm thick. Thickness was categorised into eight levels with melanomas less than 0.76 mm as a baseline group and six groupings at 1 mm increments to 6.75 mm. From 6.75 mm to 9.75 mm the numbers were rather small and were combined. This choice of categories gave regular increments across the range within which breakpoints have been reported. It also avoided having whole numbers at the margins of the intervals, which may be important as our data showed marked digit preference in reporting of thickness.

However, because these intervals are relatively large in relation to the intervals between breakpoints which previously have been described we cannot exclude, from this single analysis, the presence of breakpoints in the 0-2 mm range. Therefore, in a further analysis, cases up to 2 mm thick were studied more closely. Cases were grouped at 0.1 mm thickness intervals from 0.7 mm to 2 mm. The 0.7 mm baseline was necessary because of the small number of deaths at thinner levels. Deaths from causes other than melanoma were regarded as censored at the date of death.

Cox's proportional hazards regression model was used for survival analysis. The hazard ratio was plotted against tumour thickness. To test whether a step function or a smooth relationship between thickness and survival was most likely the following test was done for each thickness range separately. The deviance was calculated with thickness categorised and entered into the model as a factored variable. The mean thickness for each factor level was then calculated and substituted for the ordinal factor level. This term was then entered as a regressor and the deviance calculated for this model. The test is to subtract the deviance derived from the first model from the deviance derived from the second. The residual deviance gives a $\chi^2$ test on the difference in the degrees of freedom. In a separate model thickness was also used in the original units and entered as a regressor with several orders of polynomial. Estimates of 5-year survival rate for sub-groups were made with the Kaplan-Meier technique. Confidence intervals are presented where appropriate.

### Results

*Cases with Breslow thickness up to 9.75 mm thick*

The first results presented are for 997 melanomas up to 9.75 mm thick. The mean thickness was 2.50 mm (s.d. 2.02, range 0.03–9.60 mm). The male:female ratio was 296:701. The mean age was 56 years (s.d. 17.3, range 5–96 years). There were 233 deaths from melanoma and 101 deaths from other causes.

The following variables were tested: age, sex, site, histogenetic type (lentigo maligna melanoma, superficial spreading melanoma, nodular melanoma, acral lentiginous melanoma, unclassified melanoma), histological level of invasion (Clark level) and thickness. All variables were statistically significant predictors of survival in unadjusted analyses, but when adjusted for other variables only age, sex, thickness and site were significant (Table I). Level of invasion ($P = 0.305$) and histogenetic type ($P = 0.795$) were not significant. Male sex, lesions on the trunk, volar or subungual sites, increasing age and increasing thickness were associated with worse survival. A recent report has suggested that melanomas on an 'axial' site (head, neck, trunk, volar and subungual) have poorer survival than those on an extremity (all other sites) and that this is an independent predictor of survival (Clark *et al.*, 1989). This grouping was not significant in a separate adjusted analysis ($P = 0.667$). Our coding scheme did not permit grouping into BANS and non-BANS sites (Day *et al.*, 1981*b*).

If thickness is categorised as above and entered into the model as a factored variable and the hazard ratios for each thickness interval plotted at the mid-point of each interval, the hazard ratios increase as an approximately linear function of thickness with one outlying observation (deviance = 2813.263, likelihood ratio statistic = 217.031 on 13 d.f., $P < 0.001$). We suspect that the outlying observation for the 4.76–5.75 mm group is an aberrant result as subsequent points continue in a linear fashion (Figure 1). If thickness is categorised, but used as a regressor, the fit is less good (deviance = 2846.124, likelihood ratio statistic = 184.170 on 7 d.f., $P < 0.001$) and the difference is statistically significant (residual deviance = 32.861 on 6 d.f., $P < 0.001$). If the breakpoints of other authors are used, in all cases the model with thickness entered as a factor is a statistically signifiacnt better fit than when it is entered as a regressor, but no one set of breakpoints is an outstanding fit (Table II).

**Table I** Cases less than 9.75 mm thick

| | | *5-year surv.* | | *Univariate HR* | | *Multivariate HR*[a] | |
|---|---|---|---|---|---|---|---|
| | *n* | *%* | *95% CI* | *expo(β)* | *95% CI* | *expo(β)* | *95% CI* |
| Age (continuous) | 997 | – | – | 1.02 | 1.01–1.03 | 1.01 | 1.00–1.02 |
| Site Group 1 | | | | | | | |
| Head and neck | 214 | 82 | 75–87 | | | | |
| Trunk | 192 | 73 | 65–79 | 1.69 | 1.12–2.58 | 1.89 | 1.22–2.93 |
| Upper limb | 128 | 75 | 66–82 | 1.40 | 0.86–2.28 | 1.59 | 0.97–2.63 |
| Lower limb | 380 | 81 | 76–85 | 1.14 | 0.77–1.69 | 1.55 | 1.01–2.35 |
| Volar or subungual | 83 | 51 | 38–63 | 3.08 | 1.93–4.90 | 2.06 | 1.29–3.29 |
| Site Group 2 | | | | | | | |
| Head, neck, trunk volar or subungual. | 489 | 73 | 68–77 | | | | |
| Extremities | 508 | 80 | 76–83 | 0.76 | 0.59–0.99 | | |
| Sex | | | | | | | |
| Male | 296 | 66 | 60–72 | | | | |
| Female | 701 | 81 | 77–84 | 0.57 | 0.44–0.74 | 0.60 | 0.46–0.80 |
| Histogenic type | | | | | | | |
| Lent. mal. melanoma | 147 | 84 | 76–90 | | | | |
| Super. spreading | 523 | 83 | 79–86 | 1.30 | 0.80–2.10 | | |
| Nodular | 221 | 65 | 58–71 | 2.94 | 1.80–4.80 | | |
| Unclassified | 42 | 54 | 37–69 | 3.78 | 2.00–7.15 | | |
| Acral lentiginous | 64 | 60 | 45–73 | 2.94 | 1.61–5.39 | | |
| Clark's level | | | | | | | |
| II | 138 | 97 | 89–99 | | | | |
| III | 245 | 84 | 79–89 | 6.38 | 2.29–17.77 | | |
| IV | 519 | 72 | 67–76 | 12.27 | 4.54–33.16 | | |
| V | 95 | 51 | 40–62 | 22.51 | 8.06–62.83 | | |
| Thickness (mm) | | | | | | | |
| <0.76 | 219 | 95 | 90–98 | | | | |
| 0.76–1.75 | 227 | 89 | 84–93 | 3.16 | 1.49–6.70 | 3.07 | 1.45–6.53 |
| 1.76–2.75 | 182 | 79 | 72–85 | 6.23 | 3.03–12.81 | 5.73 | 2.77–11.32 |
| 2.76–3.75 | 131 | 65 | 56–73 | 10.83 | 5.30–22.14 | 9.61 | 4.68–19.76 |
| 3.76–4.75 | 100 | 58 | 46–69 | 13.40 | 6.46–27.81 | 11.95 | 5.73–24.90 |
| 4.76–5.75 | 53 | 41 | 27–55 | 26.00 | 12.27–55.09 | 22.19 | 10.37–47.47 |
| 5.76–6.75 | 35 | 51 | 31–68 | 17.83 | 7.79–40.81 | 15.86 | 6.90–36.47 |
| 6.76–9.75 | 50 | 42 | 27–56 | 25.60 | 12.04–54.42 | 22.00 | 10.25–47.20 |
| Continuous variable | 997 | – | – | 1.40 | 1.33–1.47 | 2.34 | 1.88–2.93 |
| Thickness squared | 997 | – | – | – | – | 0.94 | 0.92–0.97 |

Survival from clinical Stage 1 primary cutaneous malignant melanoma first registered in Scotland between 1979 and 1983 inclusive. Cases 0–9.75 mm thick, $n = 997$. HR = hazard ratio. [a]All results adjusted for other variables in model. Only statistically significant results are shown. Hazard ratios for sex, age and site derived from analysis using thickness factored on eight levels. Hazards ratios for thickness as a continuous variable were derived from a separate analysis.

**Figure 1** Hazard ratio by tumour thickness for primary cutaneous melanoma in Scotland registered between 1979 and 1983. Hazard ratios are plotted at the mid-point of each thickness category. Range 0–9.75 mm thick. $n = 997$.

**Figure 2** Hazard ratio by tumour thickness for primary cutaneous melanoma in Scotland registered between 1979 and 1983. Hazard ratios are plotted at the mid-point of each thickness category. Range 0–2 mm thick. $n = 520$.

**Table II** Cases less than 9.75 mm thick

| | | *5-year surv.* | | *Univariate HR* | | *Multivariate HR*[a] | |
|---|---|---|---|---|---|---|---|
| | *n* | *%* | *95% CI* | *expo(β)* | *95% CI* | *expo(β)* | *95% CI* |
| Balch breakpoints | | | | | | | |
| <0.76 | 219 | 95 | 90–98 | | | | |
| 0.76–3.99 | 558 | 80 | 76–83 | 5.90 | 3.00–11.62 | 5.42 | 2.74–10.70 |
| ⩾4.00 | 220 | 47 | 39–55 | 20.09 | 10.14–39.81 | 16.76 | 8.41–33.39 |
| Day breakpoints | | | | | | | |
| <0.85 | 245 | 95 | 90–97 | | | | |
| 0.85–1.69 | 186 | 88 | 82–92 | 2.50 | 1.27–4.94 | 2.44 | 1.23–4.82 |
| 1.70–3.60 | 316 | 74 | 69–79 | 5.96 | 3.32–10.69 | 5.33 | 2.96–9.60 |
| >3.60 | 250 | 50 | 43–57 | 14.13 | 7.94–25.14 | 12.18 | 6.81–21.79 |
| Meyskens breakpoints | | | | | | | |
| <0.85 | 245 | 95 | 90–97 | | | | |
| 0.85–1.94 | 242 | 88 | 82–91 | 2.62 | 1.37–5.01 | 2.50 | 1.31–4.79 |
| 1.95–3.99 | 290 | 72 | 66–77 | 6.68 | 3.73–11.98 | 6.02 | 3.34–10.85 |
| ⩾4.00 | 220 | 47 | 39–55 | 15.74 | 8.81–28.12 | 13.42 | 7.47–24.11 |

5-year survival rates and hazard ratios for thickness categories derived from Balch (1978), Day (1981*a*) and Meyskens (1988). Cases 0–9.75 mm thick, $n = 997$. Units of deviance removed by fitting categorised thickness as a factored variable rather than as a continuous variable:

Using Balch's breakpoints:
factor – deviance = 2840.800, LRS = 189.495 on 8 d.f., $P<0.001$
regressor – deviance = 2853.999, LRS = 176.296 on 7 d.f., $P<0.001$
residual deviance = 13.199 on 1 d.f., $P<0.001$
Using Day's breakpoints:
factor – deviance = 2834.229, LRS = 196.066 on 9 d.f., $P<0.001$
regressor – deviance = 2845.280, LRS = 185.014 on 7 d.f., $P<0.001$
residual deviance = 11.051 on 2 d.f., $P<0.01$
Using Meysken's breakpoints:
factor – deviance = 2823.613, LRS = 206.681 on 9 d.f., $P<0.001$
regressor – deviance = 2834.955, LRS = 195.340 on 7 d.f., $P<0.001$
residual deviance = 11.342 on 2 d.f., $P<0.01$

HR = hazard ratio. [a]All results adjusted for other variables in model (age, sex and site).

When thickness was fitted as a regressor in the original units (mm to two decimal places) with several orders of polynomial, there were significant linear and quadratic effects, with the hazard ratio rising in an approximately linear fashion to about 6.75 mm thick then falling with increasing thickness. However, the shape of the curve is probably excessively influenced by outliers in the higher thickness categories.

### *Cases with Breslow thickness up to 2 mm*

This interval was studied more closely to ensure that no breakpoints were missed in this range. The fit was not improved by entering thickness as a factored variable rather than as a regressor. This was true whether 0.1 mm increments were used or whether breakpoints described previously by other authors or the potentially best-fitting breakpoints (from examination of Figure 2) were entered. The distribution of the hazard ratios with thickness at 0.1 mm intervals is shown in Figure 2. Sex was not a significant variable in the adjusted analysis in this thickness range. It was not possible to examine for breakpoints below 0.7 mm because of the small number of deaths amongst thinner lesions.

### Discussion

For melanomas less than 9.75 mm thick there appears to be a continuous, probably linear, relationship between survival and thickness adjusted for sex, age and site. We hoped to unequivocally exclude the presence of breakpoints but we have not been able to do so. In all analyses over the 0–9.75 mm range thickness fits better as a factored variable than as a regressor but no one set of breakpoints has an advantage suggesting that there is no best fit. Indeed, our empirical use of eight groups also fitted better as a factored variable than as a regressor and it is unlikely that there would be as many as seven breakpoints. This suggests that a step effect is unlikely.

We made a separate study of all cases less than 2 mm thick

**Table III** Cases less than 2 mm thick

| | *n* | *5-year surv.* % | *95% CI* | *Unadjusted HR* expo(β) | *95% CI* | *Adjusted HR*[a] expo(β) | *95% CI* |
|---|---|---|---|---|---|---|---|
| Age (continuous) | 520 | – | – | 1.02 | 1.00–1.04 | 1.02 | 1.00–1.05 |
| Site Group 1 | | | | | | | |
| Head and neck | 117 | 91 | 82–95 | | | | |
| Trunk | 105 | 88 | 79–93 | 1.76 | 0.76–3.99 | 2.41 | 0.98–5.94 |
| Upper limb | 63 | 95 | 85–98 | 0.58 | 0.16–2.15 | 0.65 | 0.17–2.46 |
| Lower limb | 209 | 92 | 86–95 | 1.10 | 0.50–2.43 | 1.05 | 0.45–2.48 |
| Volar or subungual | 26 | 79 | 56–91 | 3.51 | 1.24–9.90 | 2.71 | 0.93–7.90 |
| Site Group 2 | | | | | | | |
| Head, neck, trunk volar or subungual. | 248 | 88 | 83–92 | | | | |
| Extremities | 272 | 92 | 88–95 | 0.63 | 0.36–1.09 | | |
| Sex | | | | | | | |
| Male | 133 | 86 | 79–92 | | | | |
| Female | 387 | 92 | 88–94 | 0.68 | 0.38–1.22 | | |
| Histogenic type | | | | | | | |
| Lent. mal. melanoma | 98 | 91 | 82–96 | | | | |
| Super. spreading | 342 | 92 | 88–95 | 1.24 | 0.54–2.81 | | |
| Nodular | 44 | 84 | 69–92 | 2.40 | 0.87–6.63 | | |
| Unclassified | 13 | 64 | 28–86 | 4.93 | 1.44–16.86 | | |
| Acral lentiginous | 23 | 89 | 61–97 | 1.22 | 0.25–5.87 | | |
| Clark's level | | | | | | | |
| II | 135 | 96 | 89–99 | | | | |
| III | 187 | 92 | 86–95 | 2.94 | 0.98–8.80 | | |
| IV | 194 | 85 | 78–89 | 6.36 | 2.25–17.99 | | |
| V | 4 | – | – | – | – | | |
| Thickness (mm) | | | | | | | |
| <0.70 | 205 | 96 | 91–98 | | | | |
| 0.71–0.80 | 36 | 90 | 71–97 | 3.95 | 1.25–12.44 | 4.62 | 1.43–14.91 |
| 0.81–0.90 | 28 | 89 | 69–96 | 3.70 | 0.96–14.34 | 3.63 | 0.93–14.08 |
| 0.91–1.00 | 37 | 85 | 61–95 | 3.69 | 1.08–12.62 | 3.95 | 1.14–13.65 |
| 1.01–1.10 | 19 | 88 | 61–97 | 3.55 | 0.74–17.12 | 3.60 | 0.74–17.48 |
| 1.11–1.20 | 25 | 92 | 71–98 | 2.16 | 0.45–10.41 | 2.30 | 0.47–11.17 |
| 1.21–1.30 | 22 | 91 | 68–89 | 2.67 | 0.55–12.83 | 2.31 | 0.48–11.19 |
| 1.31–1.40 | 17 | 88 | 61–97 | 6.63 | 1.94–22.66 | 8.13 | 2.31–28.64 |
| 1.41–1.50 | 19 | 95 | 68–99 | 1.81 | 0.22–14.70 | 1.42 | 0.17–11.62 |
| 1.51–1.60 | 17 | 75 | 46–90 | 9.04 | 2.64–30.93 | 9.18 | 2.58–32.61 |
| 1.61–1.70 | 18 | 89 | 62–97 | 5.46 | 1.41–21.13 | 5.99 | 1.53–23.47 |
| 1.71–1.80 | 29 | 86 | 66–94 | 4.40 | 1.29–15.03 | 3.99 | 1.16–13.79 |
| 1.81–1.90 | 12 | 92 | 54–99 | 2.73 | 0.34–22.20 | 1.95 | 0.24–16.15 |
| 1.91–2.00 | 36 | 76 | 57–87 | 9.05 | 3.44–23.81 | 8.09 | 3.05–21.45 |
| Continuous variable | 520 | – | – | 2.80 | 1.72–4.56 | 2.52 | 1.55–4.09 |

Survival from clinical Stage 1 primary cutaneous malignant melanoma first registered in Scotland between 1979 and 1983 inclusive. Cases 0–2 mm thick, *n* = 520. HR = hazard ratio. [a]All results adjusted for other variables in model. Only statistically significant results are shown. Hazard ratios for age and site derived from analysis using thickness factored in eight levels. Hazards ratios for thickness as a continuous variable were dervied from a separate analysis.

using fine 0.1 mm increments to ensure that there were no breakpoints in that range which might have been overlooked. It was not possible to use a baseline group less than 0.7 mm thick as although there were seven deaths under 0.5 mm there were no deaths in the 0.5–0.7 mm range so these cases logically have to be grouped with the thinner lesions. We cannot, therefore, exclude the presence of a breakpoint in the 0.5–0.7 mm range. All the analyses done in this range showed that thickness fitted as well as a regressor as it did as a factored variable. The results, therefore, indicate that there are no breakpoints in the 0.7–2 mm range.

The analyses of other authors (Balch *et al.*, 1978; Day *et al.*, 1981*a*; Meyskens *et al.*, 1988) have all had drawbacks. The sample sizes have been relatively small and the distribution of thickness in all series is grossly skewed towards thin tumours. Categorisation by thickness into small subgroups was likely to lead to very small numbers in some groups, particualrly in thicker categories. In the analyses used it was inevitable that breakpoints would be found due to the nature of the techniques, but these may, however, be statistical artefacts.

In conclusion, clinicians will doubtless continue to find it useful to group patients for prognostic purposes but there is no biological reason to believe that there are natural breakpoints and our results do not support the view that they exist. We think that the risk of death is probably a linear function of tumour thickness and the onus should be on the proponents of natural breakpoints to provide better biological and statistical evidence for their existence.

We are grateful to the pathologists and clinicians of the Scottish Melanoma Group for their cooperation and to the following people for statistical advice: Clive Osmond, Marie Cruddas and Carol Wickham of the Medical Research Council Environmental Epidemiology Unit, Southampton, and Ruth Pickering, the Department of Medical Statistics and Computing, Southampton General Hospital. The Scottish Melanoma Group is funded by Cancer Research Campaign grant SP1382.

**Table IV** Cases less than 2 mm thick

| | | 5-year surv. | | Univariate HR | | Multivariate HR[a] | |
|---|---|---|---|---|---|---|---|
| | $n$ | % | 95% CI | expo(β) | 95% CI | expo(β) | 95% CI |
| 'Best-fit' breakpoints | | | | | | | |
| <0.81 | 241 | 95 | 91–97 | | | | |
| 0.81–1.40 | 148 | 89 | 81–93 | 2.48 | 1.81–5.19 | 2.42 | 1.15–5.10 |
| 1.41–2.00 | 131 | 84 | 76–89 | 4.04 | 2.01–8.12 | 3.50 | 1.72–7.11 |
| Day breakpoints | | | | | | | |
| <0.85 | 245 | 95 | 90–97 | | | | |
| 0.85–1.69 | 186 | 88 | 82–92 | 2.55 | 1.29–5.03 | 2.50 | 1.26–4.97 |
| 1.70–2.00 | 89 | 83 | 73–90 | 3.72 | 1.79–7.73 | 3.16 | 1.50–6.62 |
| Meyskens breakpoints | | | | | | | |
| <0.85 | 245 | 95 | 90–97 | | | | |
| 0.85–1.94 | 242 | 88 | 82–91 | 2.65 | 1.39–5.07 | 2.50 | 1.30–4.81 |
| 1.95–2.00 | 33 | 79 | 60–90 | 4.85 | 2.01–11.72 | 4.25 | 1.75–10.32 |

5-year survival rates and hazard ratios for thickness categories dervied from Day (1981*a*) and Meyskens (1988) together with the visually best fit from Figure 2. Cases 0–2 mm thick, $n$ = 520. Units of deviance removed by fitting categorised thickness as a factored variable rather than as a continuous variable:

Using 'best fit' breakpoints:
factor – deviance = 581.032, LRS = 29.028 on 7 d.f., $P<0.001$
regressor – deviance = 581.862, LRS = 28.198 on 6 d.f., $P<0.001$
residual deviance = 0.830 on 1 d.f., $P>0.25$.
Using Day's breakpoints:
factor – deviance = 583.013, LRS = 27.046 on 7 d.f., $P<0.001$
regressor – deviance = 584.260, LRS = 25.800 on 6 d.f., $P<0.001$
residual deviance = 1.247 on 1 d.f., $P>0.25$.
Using Meysken's breakpoints:
factor – deviance = 581.910, LRS = 28.150 on 7 d.f., $P<0.001$
regressor – deviance = 582.026, LRS = 28.033 on 6 d.f., $P<0.001$
residual deviance = 0.116 on 1 d.f., $P>0.5$.
HR = hazard ratio. [a]All results adjusted for age and site.

### References

BALCH, C.M., MURAD, T.M., SOONG, S.-J., INGALLS, A.L., HALPERN, N.B. & MADDOX, W.A. (1978). A multifactorial analysis of melanoma: prognostic histopathological features comparing Clark's and Breslow's staging methods. *Ann. Surg.*, **188,** 732.

BRESLOW, A. (1970. Thickness, cross-sectional area and depth of invasion in the prognosis of cutaneous melanoma. *Ann. Surg.*, **132,** 902.

BONETT, A., RODER, D. & ESTERMAN, A. (1986). Melanoma case survival rates in South Austrialia by histological type, thickness and level of tumour at diagnosis. *Med. J. Aust.*, **144,** 680.

CLARK, W.H., ELDER, D.E., GUERRY, D., IV & 5 others (1989). Model predicting survival in stage 1 melanoma based on tumor progression. *J. Natl Can. Inst.*, **81,** 1893.

DAY, C.L., LEW, R.A., MIHM, M.C. & 4 others (1981*a*). The natural break points for primary-tumor thickness in clinical stage I melanoma. *N. Engl. J. Med.*, **305,** 1155.

DAY, C.L. Jr, SOBER, A.J., KOPF, A.W. & ? others (1981*b*). A prognostic model for clinical stage 1 melanoma of the upper extremity: the importance of anatomic subsites in predicting recurrent disease. *Ann. Surg.*, **193,** 436.

MEYSKENS, F.L. Jr, BERDEAUX, D.H., PARKS, B., TONG, T., LOESCHER, L. & MOON, T.E. (1988). Cutaneous malignant melanoma (Arizona Cancer Center Experience). 1. Natural history and prognostic factors influencing survival in patients with stage 1 disease. *Cancer*, **62,** 1207.

SONDERGAARD, K. & SCHOU, G. (1985). Survival with primary cutaneous malignant melanoma, evaluated from 2012 cases. A multivariate regression analysis. *Virchows Arch.*, **406,** 179.