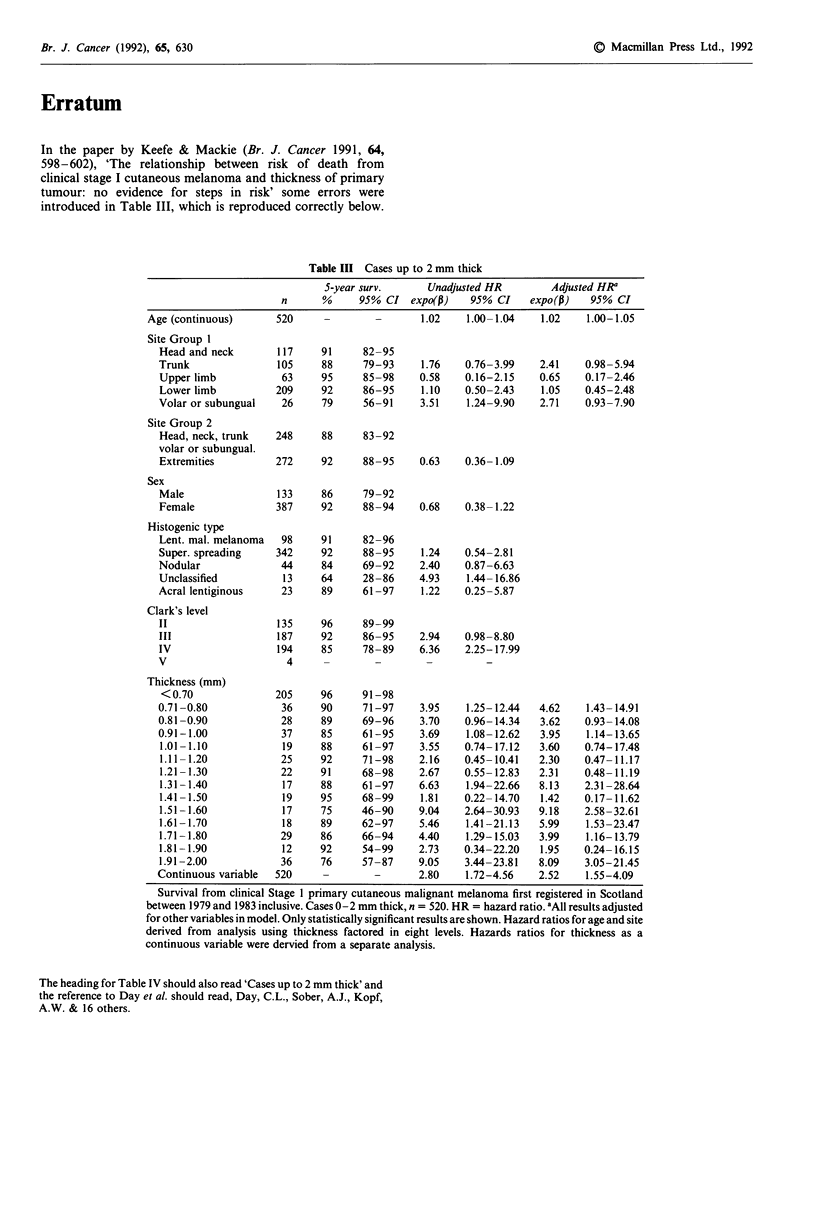# Erratum

**Published:** 1992-04

**Authors:** 


 © Macmillan Press Ltd., 1992

### Erratum

In the paper by Keefe & Mackie (*Br. J. Cancer* 1991, **64**, 598–602), 'The relationship between risk of death from clinical stage I cutaneous melanoma and thickness of primary tumour: no evidence for steps in risk' some errors were introduced in Table III, which is reproduced correctly below.

**Table III** Cases up to 2 mm thick

| | | *5-year surv.* | | *Unadjusted HR* | | *Adjusted HR*[a] | |
|---|---|---|---|---|---|---|---|
| | *n* | *%* | *95% CI* | *expo(β)* | *95% CI* | *expo(β)* | *95% CI* |
| Age (continuous) | 520 | – | – | 1.02 | 1.00–1.04 | 1.02 | 1.00–1.05 |
| Site Group 1 | | | | | | | |
| Head and neck | 117 | 91 | 82–95 | | | | |
| Trunk | 105 | 88 | 79–93 | 1.76 | 0.76–3.99 | 2.41 | 0.98–5.94 |
| Upper limb | 63 | 95 | 85–98 | 0.58 | 0.16–2.15 | 0.65 | 0.17–2.46 |
| Lower limb | 209 | 92 | 86–95 | 1.10 | 0.50–2.43 | 1.05 | 0.45–2.48 |
| Volar or subungual | 26 | 79 | 56–91 | 3.51 | 1.24–9.90 | 2.71 | 0.93–7.90 |
| Site Group 2 | | | | | | | |
| Head, neck, trunk volar or subungual. | 248 | 88 | 83–92 | | | | |
| Extremities | 272 | 92 | 88–95 | 0.63 | 0.36–1.09 | | |
| Sex | | | | | | | |
| Male | 133 | 86 | 79–92 | | | | |
| Female | 387 | 92 | 88–94 | 0.68 | 0.38–1.22 | | |
| Histogenic type | | | | | | | |
| Lent. mal. melanoma | 98 | 91 | 82–96 | | | | |
| Super. spreading | 342 | 92 | 88–95 | 1.24 | 0.54–2.81 | | |
| Nodular | 44 | 84 | 69–92 | 2.40 | 0.87–6.63 | | |
| Unclassified | 13 | 64 | 28–86 | 4.93 | 1.44–16.86 | | |
| Acral lentiginous | 23 | 89 | 61–97 | 1.22 | 0.25–5.87 | | |
| Clark's level | | | | | | | |
| II | 135 | 96 | 89–99 | | | | |
| III | 187 | 92 | 86–95 | 2.94 | 0.98–8.80 | | |
| IV | 194 | 85 | 78–89 | 6.36 | 2.25–17.99 | | |
| V | 4 | – | – | – | – | | |
| Thickness (mm) | | | | | | | |
| <0.70 | 205 | 96 | 91–98 | | | | |
| 0.71–0.80 | 36 | 90 | 71–97 | 3.95 | 1.25–12.44 | 4.62 | 1.43–14.91 |
| 0.81–0.90 | 28 | 89 | 69–96 | 3.70 | 0.96–14.34 | 3.62 | 0.93–14.08 |
| 0.91–1.00 | 37 | 85 | 61–95 | 3.69 | 1.08–12.62 | 3.95 | 1.14–13.65 |
| 1.01–1.10 | 19 | 88 | 61–97 | 3.55 | 0.74–17.12 | 3.60 | 0.74–17.48 |
| 1.11–1.20 | 25 | 92 | 71–98 | 2.16 | 0.45–10.41 | 2.30 | 0.47–11.17 |
| 1.21–1.30 | 22 | 91 | 68–98 | 2.67 | 0.55–12.83 | 2.31 | 0.48–11.19 |
| 1.31–1.40 | 17 | 88 | 61–97 | 6.63 | 1.94–22.66 | 8.13 | 2.31–28.64 |
| 1.41–1.50 | 19 | 95 | 68–99 | 1.81 | 0.22–14.70 | 1.42 | 0.17–11.62 |
| 1.51–1.60 | 17 | 75 | 46–90 | 9.04 | 2.64–30.93 | 9.18 | 2.58–32.61 |
| 1.61–1.70 | 18 | 89 | 62–97 | 5.46 | 1.41–21.13 | 5.99 | 1.53–23.47 |
| 1.71–1.80 | 29 | 86 | 66–94 | 4.40 | 1.29–15.03 | 3.99 | 1.16–13.79 |
| 1.81–1.90 | 12 | 92 | 54–99 | 2.73 | 0.34–22.20 | 1.95 | 0.24–16.15 |
| 1.91–2.00 | 36 | 76 | 57–87 | 9.05 | 3.44–23.81 | 8.09 | 3.05–21.45 |
| Continuous variable | 520 | – | – | 2.80 | 1.72–4.56 | 2.52 | 1.55–4.09 |

Survival from clinical Stage 1 primary cutaneous malignant melanoma first registered in Scotland between 1979 and 1983 inclusive. Cases 0–2 mm thick, $n = 520$. HR = hazard ratio. [a]All results adjusted for other variables in model. Only statistically significant results are shown. Hazard ratios for age and site derived from analysis using thickness factored in eight levels. Hazards ratios for thickness as a continuous variable were dervied from a separate analysis.

The heading for Table IV should also read 'Cases up to 2 mm thick' and the reference to Day *et al.* should read, Day, C.L., Sober, A.J., Kopf, A.W. & 16 others.